# Ultrasound-Guided Deep Parasternal Intercostal Plane Block in Off-Pump Cardiac Arterial Bypass Surgery: A Retrospective Cohort Single Center Study

**DOI:** 10.3390/jcm14134756

**Published:** 2025-07-04

**Authors:** Kristian-Christos Ngamsri, Roman Tilly, Sabine Hermann, Christian Jörg Rustenbach, Medhat Radwan, Eckhard Schmid, Christophe Charotte, Lina Maria Serna-Higuita, Harry Magunia

**Affiliations:** 1 Department of Anesthesiology and Intensive Care Medicine, University Hospital of Tübingen, 72076 Tübingen, Germany; roman.tilly@med.uni-tuebingen.de (R.T.); sabine.hermann@med.uni-tuebingen.de (S.H.); eckhard.schmid@med.uni-tuebingen.de (E.S.); christophe.charotte@med.uni-tuebingen.de (C.C.); harry.magunia@med.uni-tuebingen.de (H.M.); 2 Department of Thoracic, Vascular, and Cardiac Surgery, University Hospital of Tübingen, 72076 Tübingen, Germany; christian.rustenbach@med.uni-tuebingen.de (C.J.R.); medhat.radwan@med.uni-tuebingen.de (M.R.); 3 Institute of Clinical Epidemiology and Applied Biometry, Eberhard Carl University of Tübingen, 72076 Tübingen, Germany; lina.serna-higuita@med.uni-tuebingen.de

**Keywords:** cardiac surgery, parasternal intercostal plane block parasternal block, enhanced recovery after cardiac surgery, acute pain medicine, peripheral regional anesthesia

## Abstract

**Background/Objectives**: Sternal pain after cardiac surgery results in considerable discomfort and may contribute to the development of chronic postoperative sternal pain syndrome. Parasternal intercostal plane blocks have been shown to improve postoperative sternal pain and can be an essential part of enhanced recovery after cardiac surgery (ERACS). This cohort study evaluated the postoperative impact of a single-shot deep parasternal intercostal plane block (PIPB) on the requirement of analgesic medication and pain sensation up to 48 h. **Methods**: This retrospective single-center analysis evaluates the postoperative acute pain in 157 patients undergoing off-pump coronary artery bypass (OPCAB) with median sternotomy. The additive analgesic effects of deep PIPB (38 patients) were compared to a group with standard therapy but without PIPB (119 patients). To strengthen the findings, a propensity score matching analysis was performed. Outcomes included the consumption of emergency pain agents (piritramide), the requirement of the total morphine equivalent (ME), time to extubation, and ICU length of stay. Furthermore, we examined pain sensation with evaluation by using the behavioral pain score (BPS) and numeric rating score (NRS) up to 48 h after extubation. **Results**: The deep PIPB reduced the piritramide administration 24 h and 48 h after OPCAB surgery. Moreover, the requirement of ME was 24 h and 48 h after bypass surgery also significantly decreased. The one-to-one propensity score matching confirmed our primary findings and showed a decreased requirement for intravenous agents. Additionally, we observed a reduced time for extubation and a decreased NRS rating. However, no significant changes were observed in ICU length of stay, incidence of nausea, and vomiting. **Conclusions**: Our data suggests that an ultrasound-guided single-shot deep PIPB can be a valuable tool for a multimodal analgesic protocol on patients undergoing OPCAB surgery.

## 1. Introduction

Postoperative cardiac surgery pain can be challenging and needs a multimodal analgesic approach. Up-to-date fast-track cardiac anesthesia management aims to tackle pain sensation, reduce postoperative mechanical ventilation, and decrease the duration of intensive care unit (ICU) length of stay (LOS) [1,2]. Opioids, non-opioids, nonsteroidal anti-inflammatory drugs, and local anesthetics at the incision site were utilized, but not all pain control approaches have produced consistently satisfactory outcomes [3]. Intravenous opioid administration is still the favored option to manage acute postoperative pain. However, excessive intravenous opioid use during the intraoperative and postoperative period may result in undesirable adverse effects like sedation, prolonged ICU stay, nausea, or vomiting [2,4]. Traditional neuraxial anesthesia, such as epidural anesthesia, is mostly precluded due to anticoagulation or other rare but serious adverse effects [5].

Coronary artery bypass grafting (CABG) surgery, a mainstay treatment for coronary artery disease (CAD), can be performed either with (on-pump CABG) or without (off-pump CABG) cardiopulmonary bypass [6]. However, CABG offers a crucial improvement in the quality of life and survival of patients with CAD [7]. Despite the surgical developments and technical improvements, median sternotomy triggers acute postsurgical pain, and a subset of these patients can develop chronic post-sternotomy pain [8,9]. Almost 30% to 50% of patients after median sternotomy report pain lasting over two months, and 5% to 10% of this collective rate their pain severity using the numeric rating scale (NRS) as over five, requiring chronic opioid use [10].

Enhanced recovery of cardiac surgery (ERACS) is a multimodal approach aiming at early postoperative recovery, reduced perioperative pain sensation and mortality, and improved patient satisfaction [11,12]. Perioperative infiltration with local anesthetic agents has been used in several surgical settings with variable success in achieving intraoperative hemodynamic stability, reducing opioid consumption, and improving postoperative analgesic control [13]. Local anesthetic administration in the intercostal space under ultrasound guidance allows for the adequate delivery of drugs and minimizes bleeding complications and inadvertent administration in blood vessels [14]. Regional parasternal infiltration with local agents after sternotomy was described in 2005 for the first time [15,16]. In recent years, fascia plane chest wall blocks have gained popularity due to their simplicity using ultrasound, as well as a perceived low-complication profile [16]. Parasternal intercostal plane block (PIPB) with a local anesthetic agent has been shown to provide effective analgesia and reduces the need for opioids [17]. Recent studies have demonstrated the possible analgesic benefits of superficial and/or deep PIPB in various cardiac surgical settings on postoperative opioid consumption and ICU LOS [18,19]. The impact of deep PIPB in specific cardiac surgery settings, such as off-pump coronary artery bypass grafting (OPCAB), has not been well studied. The purpose of our retrospective study was to evaluate the efficacy of postoperative ultrasound-guided deep PIPB compared to a conventional standardized analgesic regimen in reducing postoperative pain in standard OPCAB surgery only.

## 2. Materials and Methods

### 2.1. Design and Patient Collective

**Figure 1:**
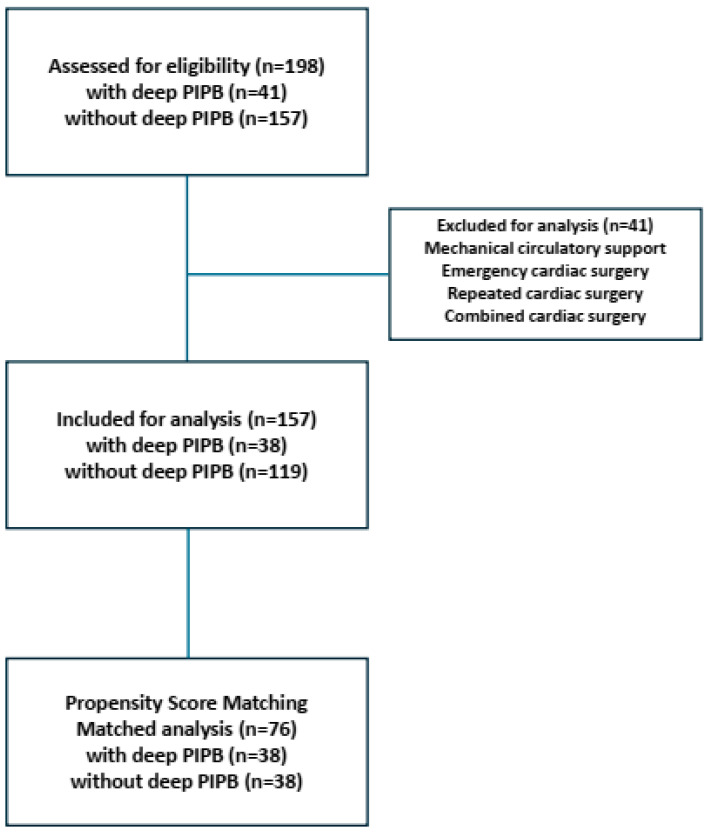
Flow diagram of patient enrollment and analysis (PIPB; parasternal intercostal plane block).

Adult patients (>18 years) undergoing elective OPCAB surgery with median sternotomy between May 2024 and December 2024 at the University Hospital Tübingen were included in this study. The study complies with the Declaration of Helsinki and has been approved by the Medical Ethics Committee of the Faculty of Medicine at the University of Tübingen. (770/2024BO2). Exclusion criteria were emergency cardiac surgery, a switch from off-pump to on-pump during cardiac surgery, the administration of extracorporeal life support, combined bypass and valve surgery, and intraoperative death. Next, patients with missing or incomplete information about the admission, procedure, and/or discharge dates, gender, and age were excluded. Furthermore, patients receiving analgesic adjuvants, like dexmetomidine or clonidine, were also excluded from further analyses. In total, 198 patients were identified and could be included in the data analysis (Figure 1).

### 2.2. Data Collection of Electronic Health Records

Electronic health records were analyzed according to the inclusion criteria. Extracted patient characteristics included age, gender, body height, body weight, body mass index, and physical status classification characterized by the American Society of Anesthesiologists (ASA) status. Furthermore, cardiovascular risk factors, like arterial hypertension, diabetes, nicotine consumption, hyperlipidemia, and obesity, were examined and used further for propensity score matching analysis.

Procedural characteristics including total surgical time (minutes), mechanical ventilation from the end of the surgery until extubation (minutes), total intensive care unit (ICU) duration (days), postoperative analgesic medication, pain numeric response scores, like the pain behavior score (PBS) and numeric rating score (NRS), nausea, including vomiting, and bleeding complications were obtained from the Patient Data Management System IntelliSpace Critical Care and Anesthesia (ICCA CC^®^; Philips; Böblingen, Germany).

### 2.3. Deep PIPB Performance and Postoperative Pain Management

The deep PIPB was performed after the completion of cardiac surgery under general anesthesia in the supine position. Experienced anesthesiologists performed the PIPB by using a high-frequency linear transducer L4/20t from GE Venue Go (GE HealthCare; Chicago, IL, USA) or 13-6 from Sono Site Edge II (FUJIFilm; Bothell, WA, USA). The sonography probe was positioned 1–2 cm lateral to the sternum, offering a parasagittal view of the parasternal chest tissue. In cranial–caudal orientation and n-plane manner, a 25-Gage needle SonoPlex^®^ (PAJUNK^®^; Geisingen, Germany) was inserted in the 2/3 and 4/5 intercostal space, with the tip reaching the deep fascia plane as described before [20]. A total of 10 mL of Ropivacain 0.375% without any adjuvant (Fresenius Kabi; Bad Homburg, Germany) was injected using a single-shot technique in each intercostal space. The same procedure was performed on the contralateral side. Following the PIPB, the patients were transferred to the ICU, where ventilation weaning and extubation was conducted according to the ICU management practices. Alternatively, they were extubated directly in the operating room and transferred to the ICU thereafter. The BPS was used to assess pain sensation in ventilated patients during the weaning period. The NRS was evaluated directly after extubation, as well as 24 h after extubation.

The ICU’s pain management regimen included a base multimodal analgesic medication consisting of oxycodone/naloxone (10–40 mg per day), pregabalin (50–100 mg per day), and magnesium (2 g per day), with the goal of achieving an NRS of less than 3. Piritramide and morphine were used intravenously as rescue analgesic agents for pain exacerbation greater than an NRS of 3. The consumption of rescue analgesic opioids and opioid requirements were recorded for each patient and calculated as total morphine equivalents (MEs), as previously described [21].

### 2.4. Outcome Endpoints and Independent Variables

The primary endpoints of this study were the cumulative consumption of rescue analgesic agents and MEs up to 48 h after OPCAB surgery. The time to extubation, ICU LOS, postoperative BPS, and NRS at rest were considered as secondary outcome points. An evaluation of adverse side effects, such as hematoma, pneumothorax, or local infection related to deep PIPB, were recorded as safety outcomes.

### 2.5. Statistical Analysis

Descriptive statistics: Continuous variables are reported as the mean and standard deviation or median and interquartile range according to their distribution. The distribution was evaluated by investigating kurtosis, skewness as Q-Q plots, and histograms. Data was be presented as dot and box plots. Categorical variables are reported as absolute and relative frequency.

Propensity score (PS) matching was used to minimize the bias resulting from differences in baseline characteristics between groups. The balance of possible confounders after PS matching was assessed with standardized mean differences (SMDs). The SMD was small for all covariates in all weighted populations, indicating that after PS matching, the distribution of the covariates was well balanced across groups. This process resulted in a balanced subgroup of 38 patients in each group (with and without PIPB).

Additionally, propensity score adjustment was employed in the binary logistic regression and ANCOVA models in the total cohort to establish causal effects between treatment groups. Several assumptions were tested before performing the models, including normal distribution, homoscedasticity, and homogeneity of variance. Residuals were inspected. In the case of severe deviation from normal distribution and/or homoscedasticity even after log transformation, the following non-parametrical methods were used: Kruskal–Wallis test, Mann–Whitney test, and the nonparametric analysis for longitudinal data “nparLD” (R-software; Version 4.5.1). All reported *p*-values are two sided and the level of significance in each analysis was set to 0.05. Statistical analysis was performed by using GraphPad Prism (Version 10.4.2), R statistical software version 4.4, and the program for social sciences IBM SPSS software version 29.0 (IBM, New York, NY, USA).

## 3. Results

A total of 198 patients underwent elective off-pump cardiac surgery via median sternotomy during July 2024 to December 2024. Of this total patient cohort, 41 patients were excluded due to extracorporeal life support utilization, switching from off-pump to on-pump surgery with or without cardioplegia or being admitted to the hospital as an emergency case.

Overall, 157 patients were analyzed and separated into two groups with and without deep PIPB. A total of 119 patients underwent the standard postoperative analgesic protocol without a deep PIPB, and 38 patients received a deep PIPB after completion of surgery in addition to standard care. The patient (Table 1) and procedural characteristics (Table 2) from both groups were evaluated. There were no differences in baseline characteristics between both patient groups (Table 1).

After 24 h, the total ME consumption was significantly reduced in the deep PIPB group compared to the control group. Forty-eight hours after OPCAB surgery, the total ME requirement was lower in both groups, but the PIPB group had significantly less total pain killer consumption than the patients without a deep PIPB. A reduced consumption of the emergency pain killer piritramide was observed 24 h after surgery in the PIPB group. Similarly, the postoperative requirement of an emergency pain agent, piritramide, was decreased 48 h after surgery in patients who had received a PIPB. No differences between the two groups were observed regarding the incidence of nausea or vomiting (Table 2). Furthermore, bleeding or respiratory complications, like pneumonia or pneumothorax, related to PIPB were not recorded in any of the patients. Side effects like opioid-related nausea and vomiting were not affected by PIPB administration (Table 1).

During weaning time, the bilateral deep PIPB was not superior to the control group without PIPB based on BPS. Interestingly, the pain evaluation with NRS directly after extubation showed no difference between the deep PIPB and the control group. An improvement of the NRS was detected 24 h after extubation; thus, the PIPB group had a significantly reduced NRS compared to the control cohort without PIPB. The positive effects of deep PIPB on NRS persisted up to 48 h after extubation, as the pain peak may already have subsided (Table 2).

**Figure 2:**
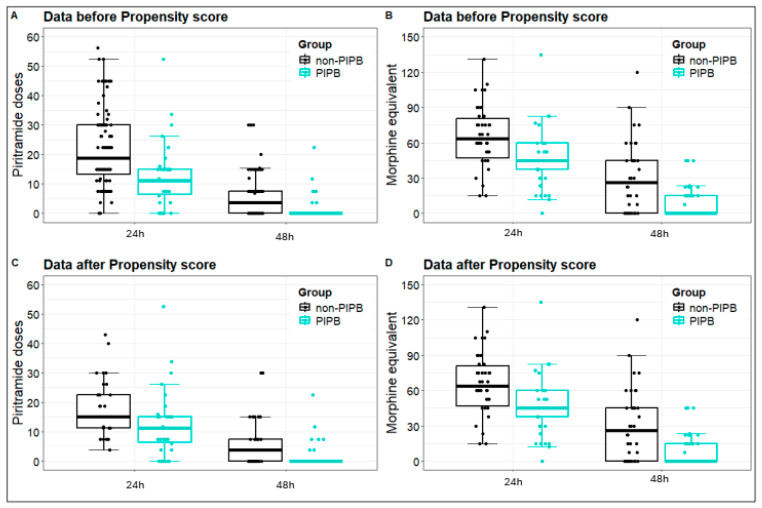
Box plot piritramide and ME doses before and after propensity score. Variables are indicated by using mean ± standard deviation. Abbreviations: PIPB: parasternal intercostal plane block; h: hours.

Next, propensity score matching was performed after the stratification of the baseline characteristics of patients with and without PIPB (Supplemental Table S1). The propensity score matching analysis confirmed our previous findings, namely that a bilateral single-shot deep PIPB reduced the postoperative consumption of piritramide 24 and 48 h after OPCAB surgery. Identically, we observed a decreased requirement of MEs in the PIPB group compared to the control group in the first and second postoperative day (Figure 2 and Table 3). To strengthen our findings after propensity score matching and in light of the small patient groups, we performed an additional analysis of covariance, which confirmed our previous results (Supplemental Table S2).

As a secondary outcome measure, we assessed the impact of deep PIPB on extubation time and ICU LOS after the propensity score matching. The bilateral parasternal blockade did not affect the ICU LOS, but the time to extubation was significantly lower in the PIPB group, confirming our unmatched data (Table 4).

## 4. Discussion

Postoperative analgesia following cardiac surgery with median sternotomy has negative effects on patients’ comfort and significantly impacts postoperative cardiac surgical outcomes [1]. In the last ten years, intense focus has been set on enhanced recovery protocols and multimodal analgesia regimens to improve outcomes in cardiac surgery [22,23]. Regional anesthesia is a crucial part of ERACS protocols, which aim to reduce opioid consumption, reduce opioid adverse effects, and improve postoperative outcomes [24]. The deep PIPB is a fascia plane block that aims to block the anterior cutaneous branches, which are responsible for sternal pain transmission [15]. Since 2005, various retrospective and prospective studies have been performed to evaluate the impact of PIPB on cardiac surgery [25,26]. After promising literature was published in 2023, we decide to first implement the deep PIPB in our hospital [20,27]. Meanwhile, depending on the cardiac surgery procedure, anatomical conditions, and anesthesiologist’s experience, both superficial and deep PIPB are performed in our department. According to the current literature, deep and superficial PIPB appear to have similar analgesic effects [28]. The PIPB is most commonly performed as a bilateral single-shot block and, in most studies prior to surgical incision, aims to reduce intraoperative opioid requirements [18,27,29]. Recent studies also suggest the benefits of a continuous administration of local anesthetics via a catheter during cardiac surgery [30,31]. To enhance our ERACS procedures, we have focused on the postoperative consumption of emergency analgesic medication and total opioid morphine equivalents by evaluating the impact of deep PIPB on postoperative pain sensation after median sternotomy. Furthermore, previous data report positive effects of a parasternal blockade on extubation time, ICU stay, and opioid-related side effects such as postoperative nausea and vomiting [14,28,32].

Our retrospective study reports a significant reduction in oral morphine equivalents 24 h and 48 h after OPCAB surgery compared to those patients who did not receive a deep PIPB. Previously, Skojec et al. confirmed the analgesic efficiency of a deep PIPB after cardiac surgery with median sternotomy, which supports our findings [33]. Wong et al. and Li et al. report in prospective randomized controlled trials intraoperative analgesic benefits of a deep PIPB but report poor effects on opioid requirements in the postoperative period [27,32]. It is important to consider the possible pharmacokinetic limitation of the pre-incisional administration of local anesthetics on postoperative pain sensation in both studies. Previous studies have observed possible positive effects of PIPB on total morphine equivalent consumption after cardiac surgery, but the use of emergency analgesic agents has not been addressed in detail [20,25,29,34]. Our data reveal that the PIPB not only curtails ME consumption but also reduces the requirement for the emergency analgesic agent piritramide.

To strengthen our unmatched data, we performed a one-to-one propensity score matching analysis and an analysis of covariance. The one-to-one propensity score matching analysis of both groups confirmed our unmatched findings, highlighting the benefits of the parasternal blockade after cardiac surgery. The deep PIPB was associated with a lower total morphine equivalent requirement 24 h and 48 h after OPCAB surgery. Chen et al. demonstrated a sustained analgesic benefit for patients who had received a PIPB in a large retrospective study [34]. Recently, Li et al. highlighted the superior analgesic effects of a bilateral PIPB on cardiac surgery with median sternotomy [35]. Our findings are in line with these previous data, which linked the single-shot performance of a deep PIPB to a reduced consumption of morphine equivalents [17,34]. Notably, most studies include various cardiac surgical operations, with and without cardiopulmonary bypass, and various perioperative anesthesia concepts. In our retrospective cohort, we followed a standardized analgesic ICU protocol, allowing us to assess the postoperative impact of PIPB on opioid requirements. Bloc et al. conducted a randomized, blinded, controlled analysis of the impact of PIPB on intraoperative anesthesia requirements and suggested positive effects on anesthesia and analgesia consumption, which aligns with our findings [36]. Additionally, the presented propensity score analysis reveals a reduced consumption of emergency analgesic agents, e.g., piritramide, 24 h and 48 h after OPCAB surgery. In line with these data, previous retrospective studies have demonstrated the benefits of PIPB in reducing the need for emergency analgesic agents after cardiac surgery [36,37,38].

Parasternal blockade after cardiac surgery via median sternotomy may reduce opioid consumption, but secondary endpoints like the reduction in mechanical ventilation time and reduced ICU length are inconclusive [18,39,40]. Our data demonstrate a reduced mechanical ventilation time, but ICU LOS did not differ between groups. Similarly, in a prospective randomized trial, Krishan et al. demonstrated in a small patient collective a decreased opioid requirement but also a reduced time of invasive mechanical ventilation [39]. Contrary, Lee et al. failed to report a significant reduction in ICU LOS or mechanical ventilation time after PIPB [41]. These contradictory data may be due to multifactorial causes, such as postoperative management by the ICU team or different respiratory weaning and extubation protocols. Organizational structures often have a greater impact on discharge from the ICU than patient needs do.

Our study has several limitations. First, it is retrospective, which makes it susceptible to inherent selection bias or unmeasured confounding bias, despite the introduction of propensity matching. Second, the small size of our patient collective and the single-center nature of our study are also limitations. Third, we did not include intraoperative opioid consumption in our analyses, which may can affect postoperative pain sensation and mechanical ventilation length. Regardless of our findings, prospective, multicenter, randomized, and blinded trials with a larger number of participants are needed in the future to examine the impact and possible adverse effects of deep PIPB in cardiac surgery.

## 5. Conclusions

In conclusion, a bilateral single-shot deep PIPB may reduce the consumption of emergency analgesic agents and MEs 24 h and 48 h postoperatively, providing superior effects in mechanical ventilation compared to standard analgesic therapy. However, further investigation of the PIPB in large prospective trials is necessary to ensure methodological consistency and safety profiles in ERACS protocols.

## Figures and Tables

**Table 1:** Patient and procedural characteristics.

	Without Deep PIPB (*n* = 119)	With Deep PIPB (*n* = 38)	*p*-Value
Age (years)	66.5 ± 9.3	64.7 ± 8.0	0.396 ^MWT^
Sex			
male	105 (88.2%)	33 (86.8%)	
female	14 (11.8%)	5 (13.2%)	
BMI (kg/m^2^)	27.8 ± 2.15	26.67 ± 3.17	0.756 ^MWT^
ASA			
II	41 (34.5%)	12 (31.6%)	
III	49 (41.2%)	17 (44.7%)	
IV	29 (24.3%)	9 (23.7%)	
Nausea/Vomiting	13 (10.9%)	5 (13.2%)	0.638 ^MWT^

Continuous variables are summarized as median ± interquartile (%). Abbreviations: BMI; body mass index; ASA: American Society of Anesthesiologists; MWT: Mann–Whitney test.

**Table 2:** Unmatched clinical characteristics and outcomes with and without deep PIPB.

	Without Deep DIPB (*n* = 119)	With Deep DIPB (*n* = 38)	*p*-Value Adjusted
Primary endpoints			
Morphine equivalent			
po day 1	67.5 mg (45.0; 90.0)	45.0 mg (35.6; 60.0)	0.0004 ^MWT^
po day 2	30.0 mg (7.5; 45.0)	0.0 mg (0.0; 16.8)	0.0004 ^MWT^
Piritramide consumption			
po day 1	15.0 mg (9.4; 27.1)	11.2 mg (5.4; 15.0)	0.004 ^MWT^
po day 2	3.8 mg (0.0; 7.5)	0.0 mg (0.0; 0.0)	0.0012 ^MWT^
Secondary endpoints			
BPS			
before extubation	3.7 ± 0.5	4.0 ± 0.3	0.660 ^MWT^
NRS			
after extubation	1.5 ± 2.3	1.0 ± 1.6	0.362 ^MWT^
po day 1	3.2 ± 1.9	1.5 ± 2.2	0.001 ^MWT^
po day 2	1.5 ± 2.0	0.6 ± 1.6	0.0028 ^MWT^

Values are presented as median and interquartile range ± standard deviation. Abbreviations: BPS: behavior pain score; NRS: numeric rating score; po: postoperative; MWT: Mann–Whitney test. *p*-values adjusted by Bonferroni.

**Table 3:** Endpoint doses of analgesic medication and procedural timepoints with and without PIPB (data after propensity score matching; *n* = 76).

	Non-PIPB (*n* = 38)	PIPB (*n* = 38)	*p*-Value Adjusted
Primary endpoints			
Morphine equivalent			
po day 1	63.7 mg (46.9; 80.6)	45 mg (37.5; 60.0)	0.004 ^WT^
po day 2	26.2 mg (0.0; 45)	0 mg (0.0; 15)	0.0008 ^WT^
Piritramide consumption			
po day 1	15 mg (11.2; 22.5)	11.2 mg (6.4; 15)	0.02 ^WT^
po day 2	3.7 mg (0.0; 7.5)	0 mg (0.0; 0.0)	0.012 ^WT^
Secondary endpoints			
ICU (days)	2 (2; 3)	2 (2; 3)	0.999 ^WT^
Time to extubation (minutes)	286.5 (169; 420)	60 (60; 162)	0.001 ^WT^

Values are presented as median and interquartile range. WT: Wilcoxon test. *p*-values adjusted by Bonferroni.

**Table 4:** Analysis of covariance to compare time of extubation and ICU stay by group (*n* = 157) (after log transformation and adjusted by propensity score matching).

	F Test	*p*-Value	β	95% CI	*p*-Value
Intercept			5.55		
Group PIPB (Extubation)	18.46	<0.001	−0.853	−1.165; −0.540	<0.001
Intercept			1.059		
Group PIPB (ICU stay)	0.105	0.746	0.026	−0.132; 0.184	0.746

## Data Availability

The original contributions presented in this study are included in the article/supplementary material. Further inquiries can be directed to the corresponding author.

## Supplementary Materials

The following supporting information can be downloaded at: https://www.mdpi.com/article/10.3390/jcm14134756/s1, Table S1: Baseline values before and after propensity score matching; Table S2: Binary logistic regression of piritramide and ME consumption after OPCAB surgery, adjusted by propensity score.

## Abbreviations

The following abbreviations are used in this manuscript:

ERACS	Enhanced Recovery After Cardiac Surgery
ME	Morphine Equivalent
LOS	Length of Stay
BMI	Body Mass Index
ICU	Intensive Care Unit
PIPB	Parasternal Intercostal Plane Block
OPCAB	Off-Pump Cardiac Artery Bypass
NRS	Numeric Rating Scale
BPS	Behavior Pain Scale
H	Hours
