# Supplementary material for: Ultrasound-Guided Deep Parasternal Intercostal Plane Block in Off-Pump Cardiac Arterial Bypass Surgery: A Retrospective Cohort Single Center Study
Source: J Clin Med. 2025 Jul 4;14(13):4756. doi: 10.3390/jcm14134756 (PMC12250427; doi:10.3390/jcm14134756)
Supplement: Supplementary file 1 [file jcm-14-04756-s001.zip › jcm-3683866-supplementary.pdf]

Table S1: Baseline values before and after propensity score matching

	**Before Propensity score matching**			**After Propensity score matching**		
	Non-PIPB (n=119)	PIPB (n=38)	SMD	Non-PIPB (n=38)	PIPB (n=38)	SMD
**Age** mean (SD)	66.6 (8.66)	65.9 (8.29)	-00846	66.4 (9.00)	65.9 (8.27)	0.1368
**Body weight** mean (SD)	83.9 (15.2)	82.5 (15.6)	-0.0918	83.9 (15.5)	82.5 (15.6)	-0.0911
**Body height** mean (SD)	174 (8.09)	173 (7.74)	-0.0734	174 (8.57)	173 (7.74)	-01292
**BMI** mean (SD)	27.7 (4.61)	27.5 (4.39)	-0.0547	27.6 (4.47)	27.5 (4.39)	-0.0288
**Arterial Hypertension** yes n (%)	80 (67.2%)	31 (81.6%)	-0.3702	32 (84.2%)	31 (81.6%)	0.0679
**Obesity** yes n (%)	31 (26.1%)	8 (21.1%)	0.1226	10 (26.3%)	8 (23.7%)	-0.0619
**Smoking** yes n (%)	18 (15.1%)	9 (23.7%)	-0.2013	8 (21.1%)	9 (23.7%)	-0.0619
**Diabetes** yes n (%)	31 (26.1%	12 (31.6%)	-0.11896	11 (28.9%)	12 (31.6%)	-0.0566
**Hyperlipidemia** yes n (%)	27 (22.7%)	19 (50.0%)	-0.5462	19 (50.0%)	19 (50.0%)	-0.1053
**Surgery time** mean (SD)	169 (45.6)	153 (33.1)	-0.4670	151 (37.6)	153 (33.1)	0.0732

Note: SMD; standard mean differences, BMI; body mass index, PIPB; parasternal intercostal plane block.

Table S2: Binary logistic regression of piritramide and ME consumption after OPCAB surgery, adjusted by propensity score.

	**OR**	**95% CI**	**P value**
**Outcome Piritramide 48 hours yes**			
**Group PIPB**	0.198	0.071 – 0.489	<0.001
**Outcome ME 48 hours yes**			
**Group PIPB**	0.273	0.119 – 0.614	0.002

Note: PIPB; parasternal intercostal plane block, ME; morphine equivalent
